# A Sensor-Fault-Estimation Method for Lithium-Ion Batteries in Electric Vehicles

**DOI:** 10.3390/s23187737

**Published:** 2023-09-07

**Authors:** Tianyu Lan, Zhi-Wei Gao, Haishuang Yin, Yuanhong Liu

**Affiliations:** Research Centre for Digitalization and Intelligent Diagnosis to New Energies, College of Electrical and Information Engineering, Northeast Petroleum University, Daqing 163000, China; lantytully@126.com (T.L.); nepuyhs@126.com (H.Y.); liuyuanhong@nepu.edu.cn (Y.L.)

**Keywords:** lithium-ion battery, sensor fault, fault diagnosis, fault estimation, descriptor observer

## Abstract

In recent years, electric vehicles powered by lithium-ion batteries have developed rapidly, and the safety and reliability of lithium-ion batteries have been a paramount issue. Battery management systems are highly dependent on sensor measurements to ensure the proper functioning of lithium-ion batteries. Therefore, it is imperative to develop a suitable fault diagnosis scheme for battery sensors, to realize a diagnosis at an early stage. The main objective of this paper is to establish validated electrical and thermal models for batteries, and address a model-based fault diagnosis scheme for battery sensors. Descriptor proportional and derivate observer systems are applied for sensor diagnosis, based on electrical and thermal models of lithium-ion batteries, which can realize the real-time estimation of voltage sensor fault, current sensor fault, and temperature sensor fault. To verify the estimation effect of the proposed scheme, various types of faults are utilized for simulation experiments. Battery experimental data are used for battery modeling and observer-based fault diagnosis in battery sensors.

## 1. Introduction

To achieve low carbon targets in transport, electric vehicles (EVs) are developing rapidly. EVs can be divided into battery electric vehicles (BEVs), hybrid electric vehicles (HEVs), and fuel cell electric vehicles (FCEVs) [1,2]. Lithium-ion batteries have the advantages of a high energy density, memoryless effect, and high cycle life, which have been widely used in EVs [3,4]. Lithium-ion batteries are a critical component in EVs, and the safety and reliability of lithium-ion batteries have attracted wide attention [5]. Some interesting results have been reported, including monitoring and diagnosis for batteries [6], and remaining useful life prediction for batteries [7,8]. During the operating process of the lithium-ion battery, the battery status can be monitored through the battery management system (BMS), which benefits the battery by allowing it to operate more safely and reliably, with an extension to the service life in batteries [9]. The regular operation of a BMS depends on the data collected by the sensors. Once a sensor is faulty, it will lead to incorrect measurements, which may adversely affect the BMS, and bring hazards to batteries. For instance, a faulty voltage sensor may cause the distortion of the measurement, which may result in the battery overcharging or over-discharging [10,11]. In addition, the fault of the current sensor will give rise to errors in the estimation of the state of charge (SOC) and state of health (SOH) [12]. As a result, the fault diagnosis of battery sensors in electric vehicles is essential, and needs to be fully explored.

Fault diagnosis is usually divided into hardware and software redundancy methods [13,14,15]. Software redundancy (or information redundancy) methods use information recorded in input and output data to achieve monitoring and fault diagnosis [16,17,18]. Information redundancy methods are categorized as model-based, signal-based, and knowledge-based approaches, among which model-based methods have been widely used in diagnosing lithium-ion battery sensor faults. In [19,20], the extended Kalman filter and the adaptive extended Kalman filter were used to estimate the terminal voltage of the battery cell, and the residuals between the measured voltage and estimated voltage represent indicators for diagnosis. A fault detection and isolation scheme was proposed in [21] for a lithium-ion battery management system using a nonlinear parity equation approach. Using the residual between the true SOC and estimated SOC of the battery in [22], a fault detection method was addressed for voltage and current sensors. In [23], sliding-mode, observer-based methods were proposed to diagnose voltage, current, and temperature sensor faults based on electrical and thermal dynamics in batteries. Through combining model-based and data-based methods, a diagnosis approach was presented in [24] to diagnose sensor and internal resistance faults. It was noted that most of the existing results in the literature focused on the diagnosis and isolation of lithium-ion battery sensor faults, with less emphasis on the fault estimation of sensor faults. Fault estimation has been a popular tool in fault diagnosis, and can provide rich information on faults, such as the size and shape of a fault [25,26]. Therefore, applying fault-estimation techniques for sensor faults in batteries is more interesting. It is also observed that most studies consider sensor bias faults only, without considering other types of sensor faults, such as time-varying fluctuation faults. As a result, there is a solid motivation to apply fault-estimation techniques for battery sensors subjected to various types of faults.

The contributions of this paper are summarized as follows:

(1) A second-order RC model and a two-state thermal model are developed to express the electrical and thermal characteristics of the lithium-ion battery. Actual battery data are used to validate the battery model.
(2) The descriptor proportional–derivative (PD) observer is applied to solve fault-estimation problems for battery sensors. Different types of common sensor faults are considered, including bias faults, time-varying fluctuation faults, and intermittent faults. The fault-estimation performance is demonstrated and analyzed using the PD observer.
(3) Based on the estimated system states and reconstructed sensor faults, real-time monitoring and diagnosis of the battery can be reached. When a sensor fault occurs, the estimated fault signal will deviate from the zero value, to alert to the occurrence of the fault. According to the estimated signal, one can also clearly, in real time, determine the size and shape of the sensor fault in batteries.

The rest of this paper is organized as follows: the battery model, including a second-order RC model and a two-state thermal model, is developed in Section 2. The fault-estimation algorithm is addressed in Section 3. The simulation verification is presented in Section 4. The paper ends with Section 5, with the conclusions.

## 2. Modelling for Batteries

The model must be available to the designer for a model-based diagnosis approach. Therefore, it is crucial to establish a battery model first. In this section, the battery equivalent circuit model and thermal model are introduced.

### 2.1. Second-Order RC Model

**Figure 1:**
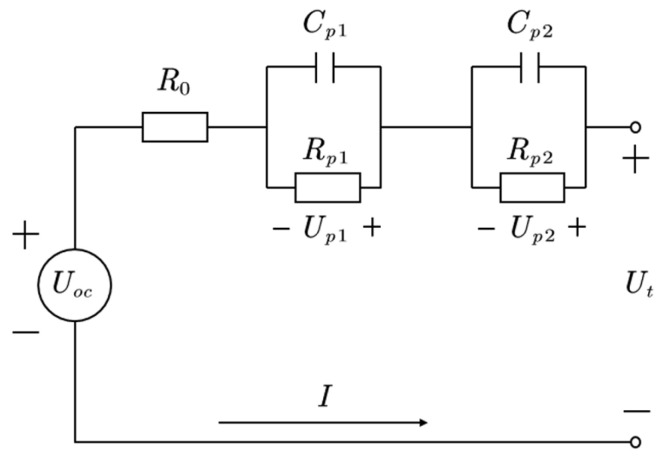
The second-order RC model of lithium-ion batteries.

The second-order RC model is used in this study, which balances the model’s accuracy and the computational demand. The model, as shown in Figure 1, is composed of an open-circuit voltage $U_{OC}$ (OCV), an ohmic resistance $R_{0}$, and two parallel RC networks ($R_{p1}$-$C_{p1}$ and $R_{p2}$-$C_{p2}$) [19]. The $R_{p1}$-$C_{p1}$ loop represents the stage of the rapid voltage change during the chemical reaction inside the battery, while the $R_{p2}$-$C_{p2}$ loop represents the stage of the slow voltage adjustment when the chemical reaction evolves inside the battery.

$U_{OC}$ is a function of the SOC. The battery SOC represents the ratio of the remaining capacity to the nominal capacity after a battery has been used or left unused for a long time. The SOC can be calculated via [22]:

(1) $$\overset{.}{SOC}=\frac{\eta_{i}}{C_{n}}I$$

where $\eta_{i}$ is the battery coulomb efficiency, $C_{n}$ is the nominal battery capacity, and *I* is the positive input current at discharge.

The relationship between $U_{p}$ and $C_{p}$ can be represented by the following formula [22]:

(2) $${\dot{U}}_{p1}=-\frac{1}{R_{1}C_{1}}U_{p1}+\frac{I}{C_{1}}$$

(3) $${\dot{U}}_{p2}=-\frac{1}{R_{2}C_{2}}U_{p2}+\frac{I}{C_{2}}$$

According to Figure 1, the terminal voltage $U_{t}$ can be calculated as follows [22]:

(4) $$U_{t}=U_{OC}+IR_{0}+U_{p1}+U_{p2}$$

and

(5) $$U_{OC}=f(SOC)$$

The relationship between $U_{OC}$ and the SOC can be obtained via the hybrid pulse power characteristic (HPPC) test, and is usually described as a nonlinear function, as shown in (5) [27]. After the discharging of the battery, the voltage will gradually become stable, which means that the chemical reactions and thermal effects inside the battery are balanced, and the battery voltage at this time is $U_{OC}$.

**Figure 2:**
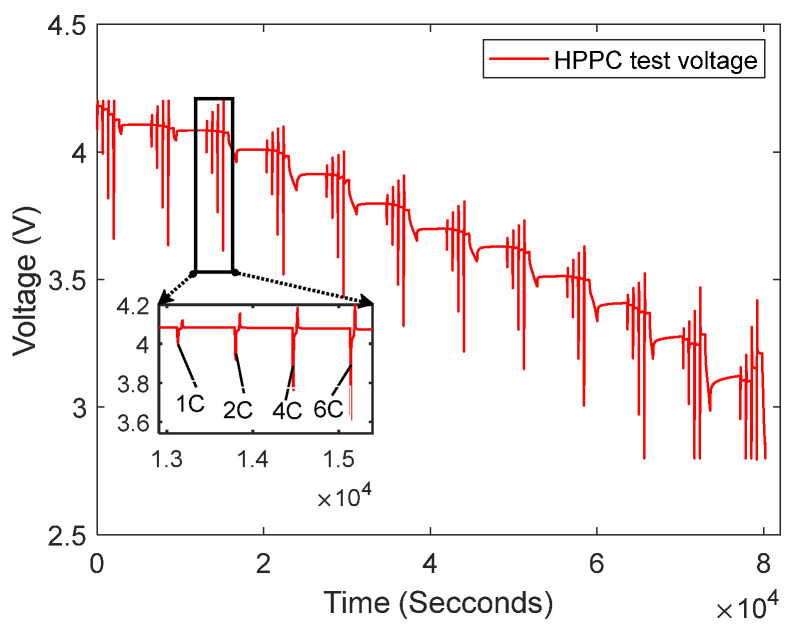
HPPC tests the voltage response curve.

We use the LG 18650HG2 lithium-ion battery data from McMaster University to obtain the relationship between $U_{OC}$ and the SOC, and to carry out the parameter identification of the second-order RC model [28]. Figure 2 shows the HPPC test voltage response curves from these data for a four-pulse discharge HPPC test at 12 different SOC values. The four pulses are 1C, 2C, 4C, and 6C discharges.

**Figure 3:**
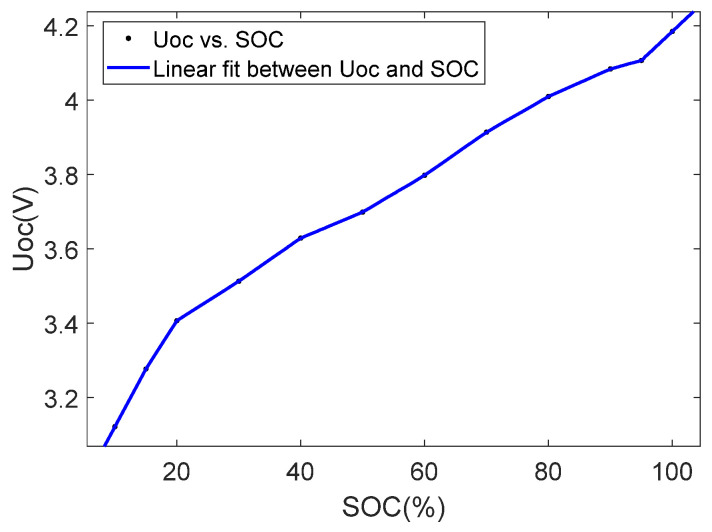
The linear fit between $U_{OC}$ and the SOC.

The relationship between $U_{OC}$ and the SOC is nonlinear. It is usually fitted as a function of higher degree. In order to simplify the relationship between the two, a gain scheduling method [29] is adopted. A known nonlinear system can be decomposed into several linear subsystems. Based on this method, the relationship between $U_{OC}$ and the SOC in the second-order RC model is divided into several parts, each of which is linear. As shown in Figure 3, the relationship between $U_{OC}$ and the SOC can be expressed as follows [29]:

(6) $$U_{OC}=a_{i}SOC_{i}+b_{i}$$

It can be seen from Figure 3 that the part of the SOC between 20% and 90% is approximately a straight line. A similar view is also presented in the paper [30]. That is, in the middle segment of the relationship between $U_{OC}$ and the SOC, it can be regarded as a linear relationship.

In summary, according to the features of Figure 3, we divide the SOC interval into four segments, and each segment has different a and b values. The parameters of the approximation of the relationship between the SOC and $U_{OC}$ are listed in Table 1.

According to the given explanation, $U_{t}$ can be described as:

(7) $$U_{t}=a_{i}SOC+U_{p1}+U_{p2}+IR_{0}+b_{i}$$

The state-space function of the second-order RC model can be rewritten as:

(8) $$\left\{\begin{array}{c} {\dot{x}}_{v}=A_{v}x_{v}+B_{v}u_{v}\\ y_{v}=C_{v}x_{v}+D_{v}u_{v} \end{array}\right.$$

where:

(9) $$A_{v}=\left(\begin{array}{ccc} 0 & 0 & 0\\ 0 & -\frac{1}{R_{1}C_{1}} & 0\\ 0 & 0 & -\frac{1}{R_{2}C_{2}} \end{array}\right)\;B_{v}=\left(\begin{array}{c} \frac{\eta_{i}}{C_{n}}\\ \frac{1}{C_{1}}\\ \frac{1}{C_{2}} \end{array}\right),C_{v}=\left(\begin{array}{ccc} a_{i} & 1 & 1 \end{array}\right),D_{v}=R_{0}\;x_{v}=\left(\begin{array}{c} SOC\\ U_{p1}\\ U_{p2} \end{array}\right),y_{v}=U_{t}-b_{i},u_{v}=I.$$

**Figure 4:**
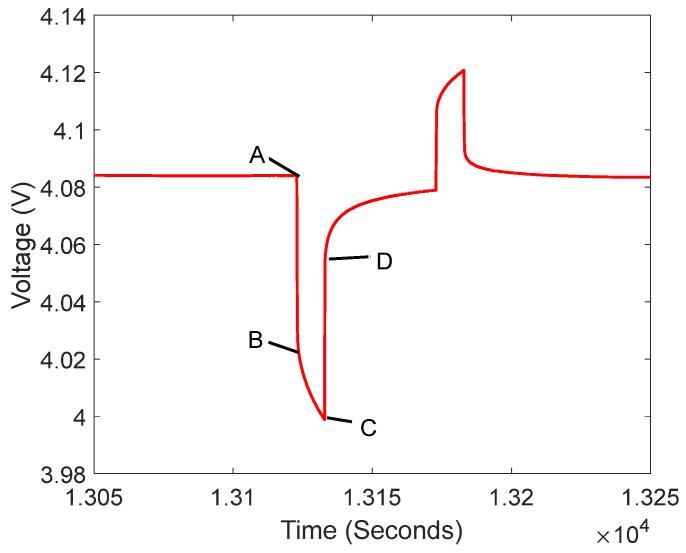
Pulse test plot at SOC = 90%, 1C discharge.

In (9), the parameters $R_{0},R_{p1},C_{p1},R_{p2},C_{p2}$ can be obtained via the HPPC test identification. Figure 4 illustrates a partial of the HPPC test shown in Figure 2; that is, the pulse test map at SOC = 90%, 1C discharge.

A, B, C, and D in Figure 4 are the points where the voltage curve changes the shape. From the second-order RC equivalent model, it can be seen that the sudden drop in voltage at the start of discharge is the effect of the internal resistance $R_{0}$, which is shown in Figure 4 for segment AB and, similarly, the rapid rise in voltage at the end of discharge is a function of $R_{0}$, which is shown in Figure 4 for the segment CD. However, the phenomenon of the gradual voltage drops during discharge, which is shown in Figure 4 for segment CD, can be explained due to the RC network. Therefore, we can obtain the value of the ohmic internal resistance $R_{0}$ of the battery from the AB and CD segments, and the value of $R_{p1},C_{p1},R_{p2},C_{p2}$ from the BC segment. Here, $R_{0}$ is calculated via (10):

(10) $$R_{0}=\frac{\left|\Delta U_{AB}\right|+\left|\Delta U_{CD}\right|}{2I}$$

The remaining four parameters can be attained via the BC segment fitting. By discretizing (4), we can obtain (11).

(11) $$U_{t}=U_{OC}+IR_{0}+IR_{p1}(1-e^{-\frac{T}{R_{p1}C_{p1}}})+IR_{p2}(1-e^{-\frac{T}{R_{p2}C_{p2}}})$$

From (11), we can have the following:

(12) $$y=a+b(1-e^{-\frac{x}{c}})+d(1-e^{-\frac{x}{f}})$$

In (12), y is the terminal voltage $U_{t}$, x is the time t. In this case, the value of x is 1. a,b,c,d,f stand for $U_{OC}+IR_{0},IR_{p1},R_{p1}C_{p1},IR_{p2},R_{p2}C_{p2}$, respectively.

The MATLAB fitting toolbox can be used to perform parameter identification for different SOC points, and to obtain the corresponding values of the above parameters under different SOCs [27].

In this study, each parameter is set as a constant value, rather than a value that changes with the SOC, through taking the arithmetic average of each parameter [23]. It is then adjusted appropriately according to the output curve measured experimentally.

According to the above modeling method, the parameters of the second-order RC model of the used LG battery are obtained, as shown in Table 2.

### 2.2. Two-State Thermal Model

**Figure 5:**
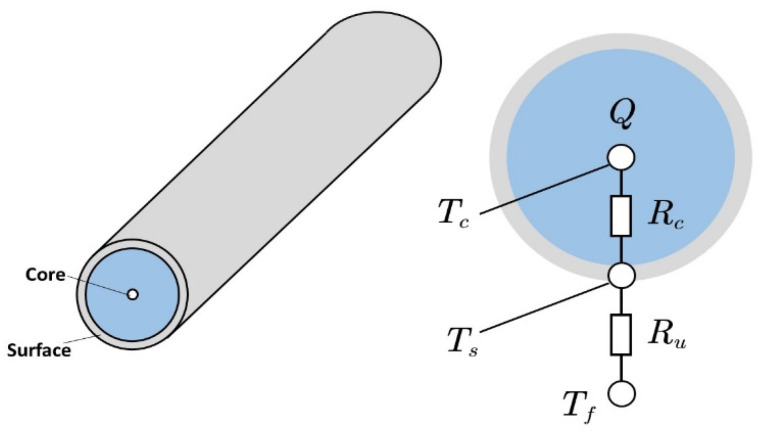
Illustration of the two-state thermal model (modified from Ref. [32]).

Through assuming longitudinal homogeneity, a two-state thermal model [31], as shown in Figure 5, is used to describe the lumped thermal dynamics of the cylindrical battery:

(13) $$\left\{\begin{array}{c} C_{c}\frac{dT_{c}}{dt}=Q-\frac{T_{c}-T_{s}}{R_{c}}\\ C_{s}\frac{dT_{s}}{dt}=\frac{T_{c}-T_{s}}{R_{u}}-\frac{T_{s}-T_{f}}{R_{c}} \end{array}\right.$$

In the equation, the state quantities $T_{s}$ and $T_{c}$ represent the battery surface and core temperature, respectively. $C_{c}$ and $C_{s}$ stand for the heat capacity of the core and of the casing, respectively. $R_{c}$ is a heat conduction resistance used to model the heat exchange between the core and the surface. $R_{u}$ is the equivalent convective resistance, which is used to simulate convective cooling on the battery surface, and $R_{u}$ is relevant to the aggregate shape of the battery package, coolant type, and coolant flow rate. Q is the amount of heat produced during the battery’s operation, which is a byproduct of the chemical reactions taking place in the electrode assembly [32]. According to Bernardi’s equation [33], the heat generated by battery Q is:

(14) $$Q=I(U_{OC}-U_{t})$$

It can be seen from (14) that the parameters of the two-state thermal model are related to the electrical model. There is a coupling between the electrical and thermal models. The heat output Q of the electrothermal model is determined by the input current  I, the open-circuit voltage $U_{OC},$ and the terminal voltage $U_{t}$ of the electrical model.

We can rewrite (13) as:

(15) $$\left\{\begin{array}{c} {\dot{x}}_{T}=A_{T}x_{T}+B_{T}u_{T}\\ y_{T}=C_{T}x_{T}+D_{T}u_{T} \end{array}\right.$$

where

(16) $$A_{T}=\left(\begin{array}{cc} -\frac{1}{R_{c}C_{c}} & \frac{1}{R_{c}C_{c}}\\ \frac{1}{C_{s}R_{c}} & -\frac{1}{C_{s}R_{c}} \end{array}\right),\;B_{T}=\left(\begin{array}{cc} \frac{1}{C_{c}} & 0\\ 0 & \frac{1}{R_{u}C_{s}} \end{array}\right),\;C_{T}=\left(\begin{array}{cc} 0 & 1 \end{array}\right),D_{T}=0,x_{T}=\left(\begin{array}{c} T_{c}\\ T_{s} \end{array}\right),y_{T}=T_{s},u_{T}=\left(\begin{array}{c} Q\\ T_{f} \end{array}\right).$$

We use a genetic algorithm to calculate the parameters. It simulates Darwin’s genetic selection in biology, the process of biological evolution via natural selection. Genetic algorithms are robust, and have a wide range of applications.

A *genetic algorithm* is a population operation that takes all individuals as the object, and mainly includes the following essential elements: (1) parameter coding, (2) initial population setting, (3) fitness function, (4) selection, (5) crossover, and (6) mutation. The genetic algorithm is used to optimize the parameters of the two-state thermal model. The primary operation process is as follows:

Step 1: Initialization. Combined with the HPPC test, the polynomial fitting relationship between $U_{OC}$ and the SOC is obtained. Moreover, the Q value is calculated according to (14). The maximum number of iterations is set to n=50, the lower boundary of the model parameter $R_{c},C_{c},R_{u},C_{s}$ search range is [1, 1, 1, 1], and the upper boundary is [10, 10, 20, 100]. Randomly generate M individuals as the initial population P(0).

Step 2: Individual evaluation. Calculate the fitness of the individuals in the population P(t), discretize Equation (15), and take the variance F of the simulated value and the actual value as the fitness function:

(17) $$F=\sqrt{\frac{{\sum_{k=1}^{k=n}\left[T_{s,m}(k)-T_{s,e}(k)\right]}^{2}}{n}}$$

Here, $T_{s,m}(k)$ represents the battery surface temperature obtained through the k*th* iteration of the model simulation, and $T_{s,e}(k)$ represents the temperature measured experimentally corresponding to the *k*th iteration.

Step 3: Select operations. The selection operator is applied to the population.

Step 4: Crossover. The crossover operator is applied to the population.

Step 5: Mutation operation. The mutation operator is applied to the population. Population *P* undergoes selection, crossover, and mutation operations to obtain the next-generation population.

**Figure 6:**
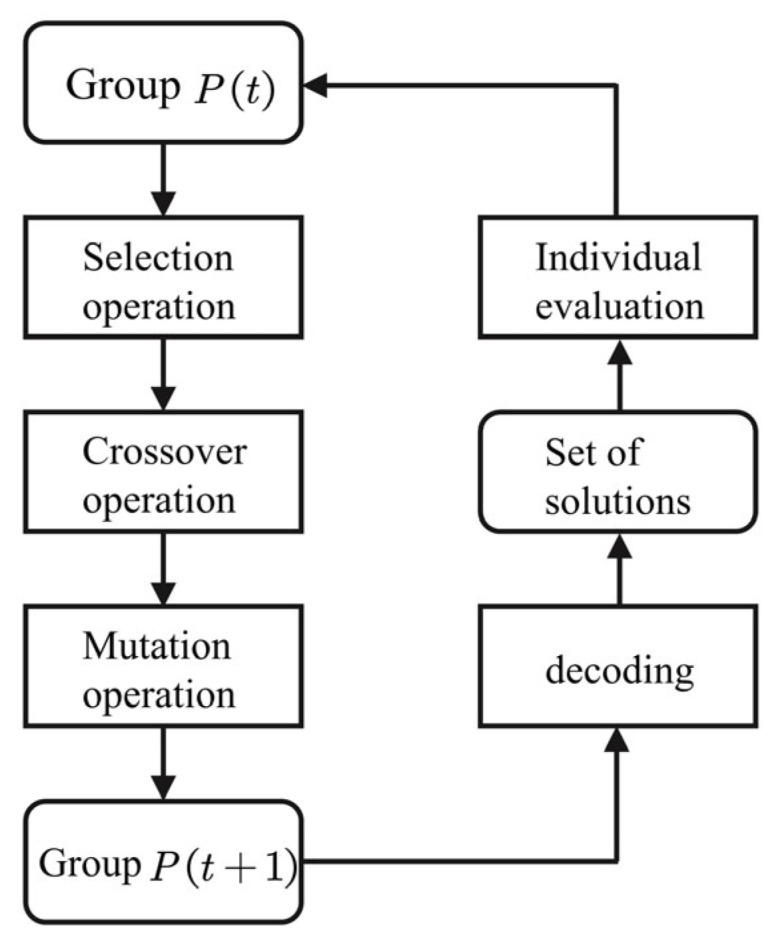
Flowchart of the genetic algorithm.

According to the above operation steps, the flow chart of the genetic algorithm is shown in Figure 6.

The algorithm identification results are shown in Table 3.

### 2.3. Fault Modeling

In the presence of a sensor fault, the output of the current, voltage, and temperature sensors can be modelled via:

(18) $$I^{m}=I+f_{I}\;U_{t}^{m}=U_{t}+f_{U}\;T_{s}^{m}=T_{s}+f_{T}$$

where $f_{I},f_{U},$ and $f_{T}$ are the faults of the corresponding sensors. It is assumed that the $f_{k}$ is bounded, where $k\in \left\{\begin{array}{ccc} I, & U, & T \end{array}\right\}$. It is also assumed that no multiple faults can occur at the same time.

To show the estimation performance of the used observer under different sensor faults, we consider applying two types of faults for the battery sensor, including the sensor bias fault [34], the sensor fluctuation fault [35], and intermittent fault. In order to intuitively see the effect of fault estimation, we use sinusoidal signals, noise signals, and intermittent signals, in turn, to simulate sensor faults.

## 3. Fault-Estimation Algorithm

Based on the above model, a modified proportional and derivative (PD) observer [25] is used to diagnose different types of faults.

In this section, we present a PD observer, to estimate the model state, and the output sensor faults at the same time. For this purpose, the following definitions are made:

(19) $$x_{\omega }(t)=f_{k}(t),\bar{x}(t)=\left(\begin{array}{c} x(t)\\ x_{\omega }(t) \end{array}\right),\bar{B}=\left(\begin{array}{c} B\\ 0 \end{array}\right),\;\bar{N}=\left(\begin{array}{c} 0\\ I_{p} \end{array}\right),\bar{E}=\left(\begin{array}{cc} I_{n} & 0\\ 0 & 0 \end{array}\right),\bar{A}=\left(\begin{array}{cc} A & 0\\ 0 & -I_{p} \end{array}\right),\;\bar{C}=\left(\begin{array}{cc} C & I_{p} \end{array}\right),{\bar{C}}_{0}=\left(\begin{array}{cc} C & 0 \end{array}\right).\;$$

where $x(t)\in R^{n}$ is the state vector, $u(t)\in R^{m}$ is the input vector, $y(t)\in R^{p}$ represents the measurement output vector, and $f_{k}(t)\in R^{p}$ is the output sensor failure vector, which is bounded.

According to (19), we can get an augmented descriptor plant [25]:

(20) $$\left\{\begin{array}{l} \bar{E}\dot{\bar{x}}(t)=\bar{A}\bar{x}(t)+\bar{B}u(t)+\bar{N}f_{k}(t)\\ y(t)={\bar{C}}_{0}\bar{x}(t)+Du\left(t\right)+x_{\omega }(t)=\bar{C}\bar{x}(t)+Du(t) \end{array}\right.$$

If, and only if, A is a stable matrix, the gain matrices $\bar{L},\bar{K}\in R^{(n+p)\times p}$ exist for the following PD observer [25]:

(21) $$\left(\bar{E}+\bar{L}\bar{C})\dot{\zeta }(t)=(\bar{A}-\bar{N}{\bar{C}}_{0}-\bar{K}\bar{C})\zeta (t)+\bar{B}u(t\right)$$

such that $\hat{\bar{x}}(t)=\zeta (t)+(\bar{E}+\bar{L}\bar{C}{)}^{-1}\bar{L}(y(t)-Du(t))$ is an asymptotic estimate of $\bar{x}(t)$ in (20).

If the model is unstable but detectable, the gain matrices ${\bar{L}}_{1},{\bar{K}}_{1}\in R^{(n+p)\times p}$ exist for the following PD observer [25]:

(22) $$\left(\bar{E}+{\bar{L}}_{1}\bar{C}){\dot{\zeta }}_{1}(t)=(\bar{A}-{\bar{K}}_{1}\bar{C})\zeta_{1}(t)+\bar{B}u(t)+\bar{A}(\bar{E}+{\bar{L}}_{1}\bar{C}{)}^{-1}{\bar{L}}_{1}(y(t)-Du(t)\right)$$

such that $\hat{\bar{x}}(t)=\zeta_{1}(t)+(\bar{E}+{\bar{L}}_{1}\bar{C}{)}^{-1}{\bar{L}}_{1}(y(t)-Du(t))$ is an asymptotic estimate of $\bar{x}(t)$ in (20).

We summarize the application method of the algorithm (Algorithm 1) in this paper via a pseudo-code, as follows:
**Algorithm 1** Select the appropriate PD observer**Input:** The state space equation of the system**Output:** The applicable form of the PD observer1:**begin**2:Form the augmented matrices of the form (19) according to the input3:Construct the descriptor plant (20).4:**if** matrix A of input is stable 5:    Using PD observer (21)6:**else if** matrix A is not stable but the system is detectable7:    Using PD observer (22)8:**end**

In this study, different PD observers are selected to estimate sensor faults, according to the stability and observability of lithium-ion battery models.

## 4. Simulation Verification and Discussion

In this section, we conduct MATLAB/Simulink simulations to verify the effect of the PD observer in estimating the fault of lithium-ion battery sensors.

**Figure 7:**
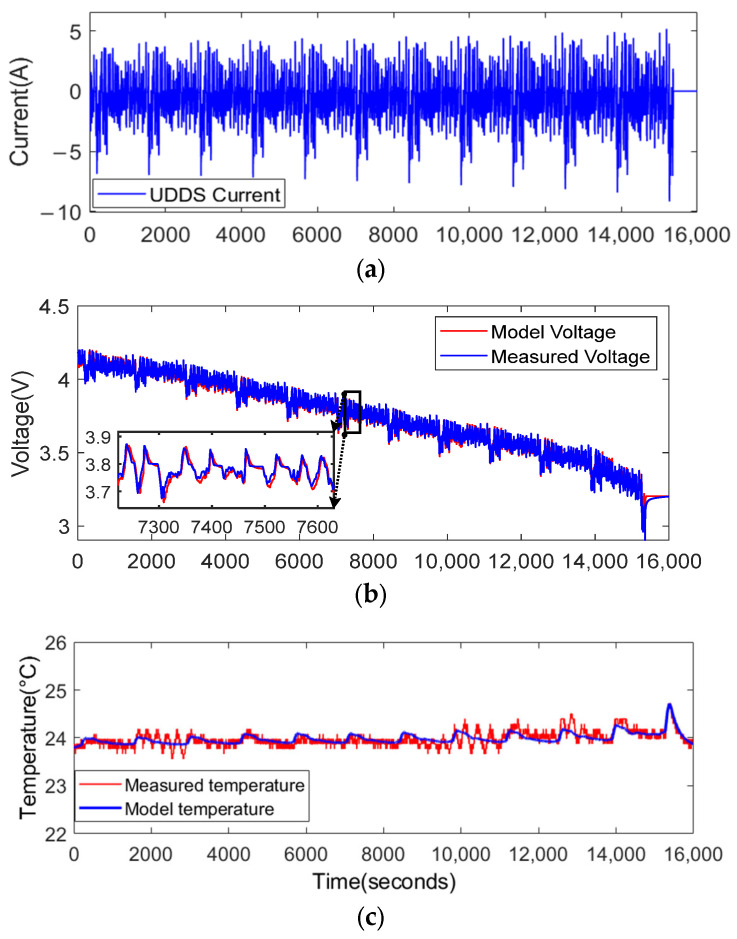
The UDDS test input current and model output versus the measured output: (**a**) the UDDS test current, (**b**) comparison of the model voltage and measured voltage, (**c**) comparison of the model output temperature and measured temperature.

The urban dynamometer driving schedule (UDDS) test at 25 °C of the LG 18650HG2 lithium-ion battery is selected to simulate the battery operating conditions of EVs. Based on the model parameters identified in Section 2 and the input of the UDDS test, the model’s output can be obtained. From Figure 7, one can see that the model output and actual output are generally consistent.

### 4.1. Fault Estimation of Lithium-Ion Battery Temperature Sensor

In this subsection, we first analyze the stability and observability of the two-state thermal model (13); the polynomial can be calculated as

(23) $$\det(\lambda I-A_{T})=\lambda^{2}+(\frac{1}{R_{c}C_{c}}+\frac{1}{R_{c}C_{s}}+\frac{1}{R_{u}C_{s}})\lambda +\frac{1}{R_{c}C_{c}R_{u}C_{s}}$$

From the well-known Routh–Hurwitz criterion, the system matrix $A_{T}$ is asymptotically stable. The observability matrix of the model (13) is given as follows:

(24) $$O_{1}=\left(\begin{array}{c} C_{T}\\ C_{T}A_{T} \end{array}\right)=\left(\begin{array}{cc} 0 & 1\\ \frac{1}{C_{s}R_{c}} & -\frac{1}{C_{s}R_{c}}-\frac{1}{C_{s}R_{u}} \end{array}\right)$$

Therefore, the battery thermal model (13) is observable.

We use a normal distributed random number $f_{k1}(t)$ with the power of 0.01, and sinusoidal signal $f_{k2}(t)=\sin(2t)$, $f_{k3}(t)=\sin(t),$ and an intermittent fault $f_{k4}(t)$ occurring at 7000 s and 14,000 s, to represent sensor fluctuation fault. In addition, we consider $f_{k5}(t)=0.5$ to denote the sensor bias fault.

Because $A_{T}$ is stable, we consider the use of observer (21) for fault diagnosis with fault estimation. Choosing:

(25) $${\bar{L}}_{T}=\left(\begin{array}{c} 0\\ 0\\ 1 \end{array}\right)$$

such that $\left(\bar{E}+{\bar{L}}_{1}\bar{C}\right)$ is nonsingular. Via this model, the following matrix can be selected:

(26) $${\bar{K}}_{T}=\left(\begin{array}{c} 2.4522\times 10^{6}\\ -3.1957\times 10^{7}\\ -0.9950 \end{array}\right)$$

**Figure 8:**
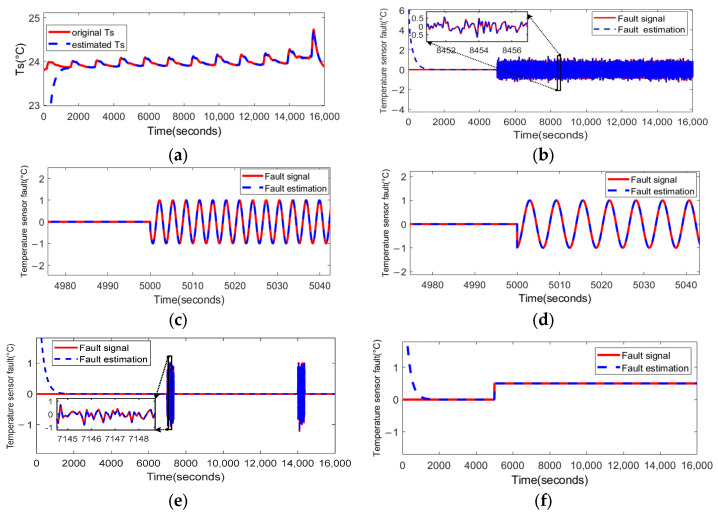
The PD observer (21) applied to the fault estimation of the temperature sensor fault: (**a**) the observation of state $T_{s}$ by observer (21) when there is no fault; (**b**) the estimated results when the time-varying fluctuation fault $f_{T1}(t)$ occurs at 5000 s; (**c**) the estimated results when the time-varying fluctuation fault $f_{T2}(t)$ occurs at 5000 s; (**d**) the estimated results when the time-varying fluctuation fault $f_{T3}(t)$ occurs at 5000 s; (**e**) the estimated results when the intermittent fault $f_{T4}(t)$ occurs at 7000 s and 14,000 s; (**f**) the estimated results when the bias fault $f_{T5}(t)$ occurs at 5000 s.

The same fault is applied to the temperature sensor, and the fault-estimation results are shown in Figure 8.

It can be seen that the observer (21) shows an excellent fault-estimation performance for all the sensor faults imposed, including biased faults, fluctuation faults, and intermittent faults.

### 4.2. Fault Estimation of Lithium-Ion Battery Current Sensor

In order to be able to design the corresponding observer for the current sensor fault $f_{I}$, the model (8) is recombined and transformed [34]:

(27) $$\left\{\begin{array}{c} {\dot{x}}_{c}(t)=A_{c}x_{c}(t)+B_{c}y_{c}(t)\\ I(t)=C_{c}x_{c}(t)+D_{c}y_{c}(t) \end{array}\right.$$

where

(28) $$A_{c}=-\frac{B_{v}C_{v}}{D_{v}}+A_{v}=\left(\begin{array}{ccc} -\frac{a_{i}\eta_{i}}{C_{n}R_{0}} & -\frac{\eta_{i}}{C_{n}R_{0}} & -\frac{\eta_{i}}{C_{n}R_{0}}\\ -\frac{a_{i}}{C_{1}R_{0}} & -\frac{1}{C_{1}R_{0}}-\frac{1}{R_{1}C_{1}} & -\frac{1}{C_{1}R_{0}}\\ -\frac{a_{i}}{C_{2}R_{0}} & -\frac{1}{C_{2}R_{0}} & -\frac{1}{C_{2}R_{0}}-\frac{1}{R_{2}C_{2}} \end{array}\right);\;B_{c}=\frac{B_{v}}{D_{v}}=\left(\begin{array}{c} \frac{\eta_{i}}{C_{n}R_{0}}\\ \frac{1}{C_{1}R_{0}}\\ \frac{1}{C_{2}R_{0}} \end{array}\right);\;C_{c}=-\frac{C_{v}}{D_{v}}=\left(\begin{array}{ccc} -\frac{a_{i}}{R_{0}} & -\frac{1}{R_{0}} & -\frac{1}{R_{0}} \end{array}\right);\;D_{c}=\frac{1}{D_{v}}=\frac{1}{R_{0}}.$$

**Figure 9:**
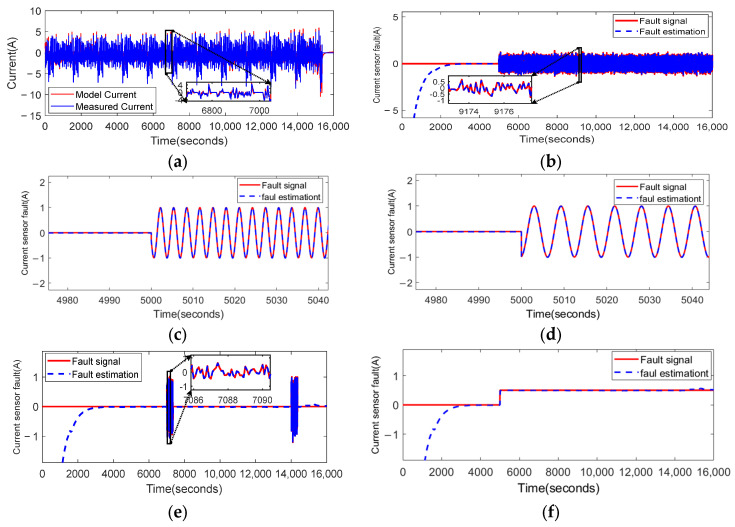
The PD observer (21) applied to the fault estimation of voltage sensor: (**a**) comparison of the model current and measured current; (**b**) the estimated results when the time-varying fluctuation fault $f_{I1}(t)$ occurs at 5000 s; (**c**) the estimated results when the time-varying fluctuation fault $f_{I2}(t)$ occurs at 5000 s; (**d**) the estimated results when the time-varying fluctuation fault $f_{I3}(t)$ occurs at 5000 s; (**e**) the estimated results when the intermittent fault $f_{I4}(t)$ occurs at 7000 s and 14,000 s; (**f**) the estimated results when the bias fault $f_{I5}(t)$ occurs at 5000 s.

The accuracy of the system (27) can be improved via adjusting the matrix $D_{v}$ appropriately. The comparison between the model output and the experimental measured output is shown in Figure 9a.

The characteristic polynomial of the matrix $A_{c}$ can be described as follows:

(29) $$\det(\lambda I-A_{c})\;=\lambda^{3}+(\frac{1}{R_{0}C_{2}}+\frac{1}{R_{2}C_{2}}+\frac{1}{R_{0}C_{1}}+\frac{1}{R_{1}C_{1}}+\frac{a_{i}\eta_{i}}{R_{0}C_{n}})\lambda^{2}\;+\left(\frac{1}{C_{1}C_{2}R_{0}R_{2}}+\frac{1}{C_{1}C_{2}R_{0}R_{1}}+\frac{1}{C_{1}C_{2}R_{1}R_{2}}+\frac{a_{i}\eta_{i}}{C_{n}C_{2}R_{0}R_{2}}+\frac{a_{i}\eta_{i}}{C_{n}C_{1}R_{0}R_{1}}\right)\lambda \;+\frac{a_{i}\eta_{i}}{C_{n}C_{1}C_{2}R_{1}R_{2}R_{0}}\;=\lambda^{3}+\beta_{2}\lambda^{2}+\beta_{1}\lambda +\beta_{0}$$

It is straightforward to verify $\beta_{2},\;\beta_{1},\;\beta_{0}>0,$ and $\beta_{2}\beta_{1}>\beta_{0}$. As a result, from the well-known Routh–Hurwitz criterion, the system matrix $A_{c}$ is asymptotically stable.

As $A_{c}$ is asymptotically stable, therefore, we can conclude that:

(30) $$\left(\begin{array}{c} sI-A_{c}\\ C_{c} \end{array}\right)$$

is full of column rank for all the complex numbers on the right closed complex plane. Therefore, the pair $\left(\begin{array}{cc} A_{c} & C_{c} \end{array}\right)$ is detectable. As a result, both observer (21) and (22) exist. Here, we only use observer (21) for our study.

We can use observer (21) for fault diagnosis with fault estimation, choosing

(31) $${\bar{L}}_{1}=\left(\begin{array}{c} 0\\ 0\\ 0\\ 1 \end{array}\right)$$

such that $\left(\bar{E}+{\bar{L}}_{1}\bar{C}\right)$ is nonsingular. Via this model, the following matrix can be selected:

(32) $${\bar{K}}_{1}=\left(\begin{array}{c} -3.1426\times 10^{8}\\ 1.1741\times 10^{8}\\ -1.6427\times 10^{7}\\ -0.5001 \end{array}\right)$$

The fault-estimation results are shown in Figure 9.

It can be seen from Figure 9 that the observer (21) shows an outstanding estimation performance for all the types of sensor faults considered: fluctuation faults, bias faults, and intermittent faults in the current sensor.

### 4.3. Fault Estimation of Lithium-Ion Battery Voltage Sensor

From $\det(\lambda I-A_{v})=0$, one can find the set of the eigenvalues to the matrix $A_{v}$ is $\left\{\begin{array}{ccc} 0, & \frac{1}{R_{1}C_{1}}, & \frac{1}{R_{2}C_{2}} \end{array}\right\}$. Therefore, the system matrix $A_{v}$ is stable, but not asymptotically stable.

The observability of the battery model (8) can be judged based on the observability matrix:

(33) $$O=\left(\begin{array}{c} C\\ CA_{v}\\ CA_{v}^{2} \end{array}\right)=\left(\begin{array}{ccc} a_{i} & 1 & 1\\ 0 & -\frac{1}{R_{1}C_{1}} & -\frac{1}{R_{2}C_{2}}\\ 0 & -\frac{1}{R_{1}^{2}C_{1}^{2}} & -\frac{1}{R_{2}^{2}C_{2}^{2}} \end{array}\right)$$

The observability matrix above is full of rank; that is, the battery RC model is observable. As a result, we can only use observer (22), rather than observer (21).

Observer (22) is used for state observation and voltage sensor fault estimation. We choose

(34) $${\bar{L}}_{v}=\left(\begin{array}{c} 0\\ 0\\ 0\\ 1 \end{array}\right)$$

such that $\left(\bar{E}+\bar{L}\bar{C}\right)$ is nonsingular. Via this model, the following matrix can be selected:

(35) $${\bar{K}}_{v}=\left(\begin{array}{c} 0.0452\\ 0.0013\\ 0.0011\\ -0.5200 \end{array}\right)$$

**Figure 10:**
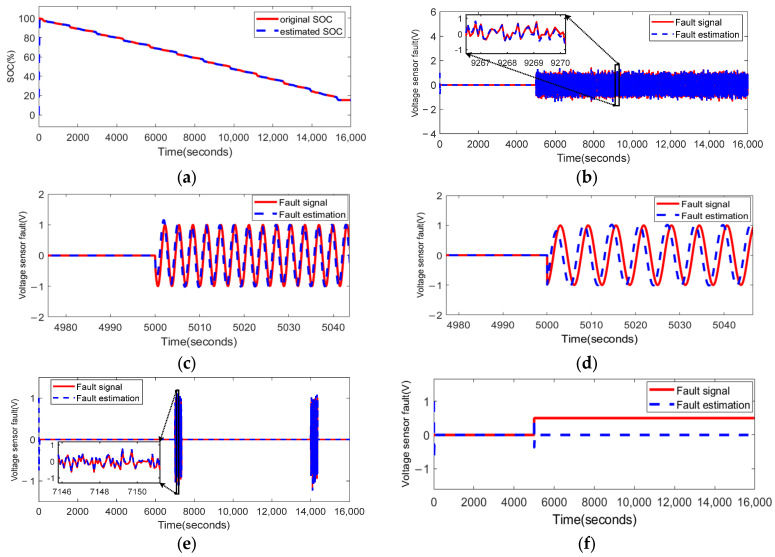
The PD observer (22) applied to the fault estimation of the voltage sensor: (**a**) the observation of the SOC by the observer when there is no fault; (**b**) the estimated results when the time-varying fluctuation fault $f_{U1}(t)$ occurs at 5000 s; (**c**) the estimated results when the time-varying fluctuation fault $f_{U2}(t)$ occurs at 5000 s; (**d**) the estimated results when the time-varying fluctuation fault $f_{U3}(t)$ occurs at 5000 s; (**e**) the estimated results when the intermittent fault $f_{U4}(t)$ occurs at 7000 s and 14,000 s; (**f**) the estimated results when the bias fault $f_{U5}(t)$ occurs at 5000 s.

Figure 10 illustrates the fault-estimation effect of the PD observer for the voltage sensor.

It can be seen from Figure 10 that the observer demonstrates an excellent fault-estimation performance on the fluctuation fault $f_{U1}(t)$ and $f_{U2}(t)$. However, for the fault $f_{U3}(t)$, with a relatively low frequency, the fault-estimation performance is slightly worse compared with high-frequency signals such as $f_{U1}(t)$ and $f_{U2}(t)$. As for the bias fault $f_{U4}(t)$, although the fault-estimation curve clearly reaches the value at the instant when the bias fault occurs, it soon fades down. It can still recognize the fault occurrence and fault size, but fails to attain the shape of the fault. Even so, the observer (22) can well-estimate a wide range of sensor faults, including high-frequency fluctuation faults and intermittent faults.

A comparison between the addressed fault-estimation approach to battery sensor faults in this study, and the existing results, is shown in Table 4.

## 5. Conclusions

Sensor fault diagnosis is of great significance to lithium-ion battery reliability and safety. For the second-order RC model and the two-state thermal model of the lithium-ion battery, the addressed sensor fault estimation can perfectly estimate both high-frequency and low-frequency sensor faults. The fault estimator can effectively estimate high-frequency sensor fault signals for the voltage sensor fault, but reluctantly reconstructs the sensor faults, with constant bias. From the viewpoint of the whole performance, the addressed sensor fault-estimation techniques can cover a wide range of sensor faults in electric batteries, which have provided a powerful tool for real-time fault diagnosis for sensors in electric batteries.

In the future, it is of interest to investigate the internal resistance fault in electric batteries, using fault-estimation techniques.

## Figures and Tables

**Table 1:** The parameters of the approximation of the relationship between $U_{OC}\;$ and the SOC.

$i^{th}$	1	2	3	4
$SOC_{i}(\%)$	0–20	20–90	90–95	95–100
$a_{i}$	2.850	1.065	1.000	1.560
$b_{i}$	2.837	3.148	3.148	2.625

**Table 2:** The parameters for the second-order RC model identification results.

**Items**	$R_{0}\;(\Omega )$	$R_{p1}\;(\Omega )$	$R_{p2\;}(\Omega )$	$C_{p1}\;(\Omega )$	$C_{p2}\;(\Omega )$
**Identification results**	0.0080	0.0083	0.0186	888.4861	292.5230

**Table 3:** The parameters for the two-state thermal model identification results.

**Items**	$R_{c}(K/W)$	$R_{u}(K/W)$	$C_{c}(J/K)$	$C_{s}(J/K)$
**Identification results**	7.5043	1.8184	25.9621	9.9664

**Table 4:** Comparison between the PD observer and other fault diagnosis methods.

Fault Diagnosis/Estimation Methods for Lithium-Ion Battery Sensors	Advantages	Limits
Unscented Kalman filter (UKF) [22]	Using the difference between the true SOC and the estimated SOC as the residual, the fault detection of the voltage sensor and the current sensor of the lithium-ion battery pack is cleverly realized.	Only fault detection and fault isolations are discussed; the fault size and shape cannot be obtained.
Luenberger observer and learning observer (LOs) [30]	The synthesis involving the Luenberger observer and LOs can simultaneously achieve fault isolation and estimation.	The algorithm is more complicated, and needs to assume $\dot{I}=0.$ The proposed method is more suitable for handling constant or slow-varying faults.
Sliding mode observer 1 [23]	Three sliding mode observers and three filters are designed, to realize fault diagnosis, isolation, and estimation in the lithium-ion battery voltage, current, and temperature sensors.	The fault estimation only considers a bias fault, which is questionable for handling high-frequency faults.
Sliding mode observer 2 [36]	The fault estimation of temperature and voltage sensors, including bias and varying faults, is implemented.	No current sensor fault is considered. The system needs to decompose into two sub-systems, which is somewhat complex to implement.
PD descriptor observer-based fault-estimation methods used in this paper	Three PD observers are used to estimate the voltage sensor fault, current sensor fault, and thermal sensor fault. It can handle a wide range of sensor faults, including high-frequency and low-frequency sensor faults, to detect, isolate, and identify the faults. Original system matrices are used to construct the observer, so that it is easy to implement.	For the voltage sensor abrupt fault, it can detect fault, and obtain the size the fault, but cannot obtain the shape of the fault.

## Data Availability

The battery data used in this paper are from the public source. No new data were created in this study.
